# Summer Courses of Lectures in Paris

**Published:** 1861-07

**Authors:** 


SUMMER COURSES OF LECTURES IN PARIS.—The following are the courses of lectures announced for the summer session at the Paris Faculty of Medicine:—

Pharmacology, by Professor Regnault: on Mondays, Wednesdays, and Fridays, at 10¾.

Materia Medica and Therapeutics, Professor Grisolle: Mondays, Wednesdays, and Fridays, at 3.

Obstetrics and Diseases of Women and Children, Professors Moreau and Pajot: Mondays, Wednesdays, and Fridays, at 2.

Physiology, Professor Longet: Mondays, Wednesdays, and Fridays, at 12.

Medical Jurisprudence, Professors Adelon and Tardieu: Mondays, Wednesdays, and Fridays, at 4.

Hygiene, Professor Bouchardat: Tuesdays, Thursdays, and Saturdays, at 4.

Natural Medical History, Professor Moquin-Tandon: Tuesdays, Thursdays, and Saturdays, at 11.

Surgical Pathology, Professor Gosselin: Tuesdays, Thursdays, and Saturdays, at 12.

Medical Pathology, Professor Monneret: Tuesdays, Thursdays, and Saturdays, at 3.

Pathological Anatomy, Professors Cruveilhier and Barth: Tuesdays, Thursdays, and Saturdays, at 2.

The Clinical Lectures at the hospitals by the same professors as in the winter, every day at nine.

